# Supplementary material for: Next-generation large-scale binary protein interaction network for Drosophila melanogaster
Source: Nat Commun. 2023 Apr 15;14:2162. doi: 10.1038/s41467-023-37876-0 (PMC10105736; doi:10.1038/s41467-023-37876-0)
Supplement: Supplementary file 1 — Supplementary Information [file 41467_2023_37876_MOESM1_ESM.pdf]

Supplementary Information

1. Supplementary Figures
2. Supplementary Tables
3. Supplementary Methods
4. Supplementary Notes

1. Supplementary Figures

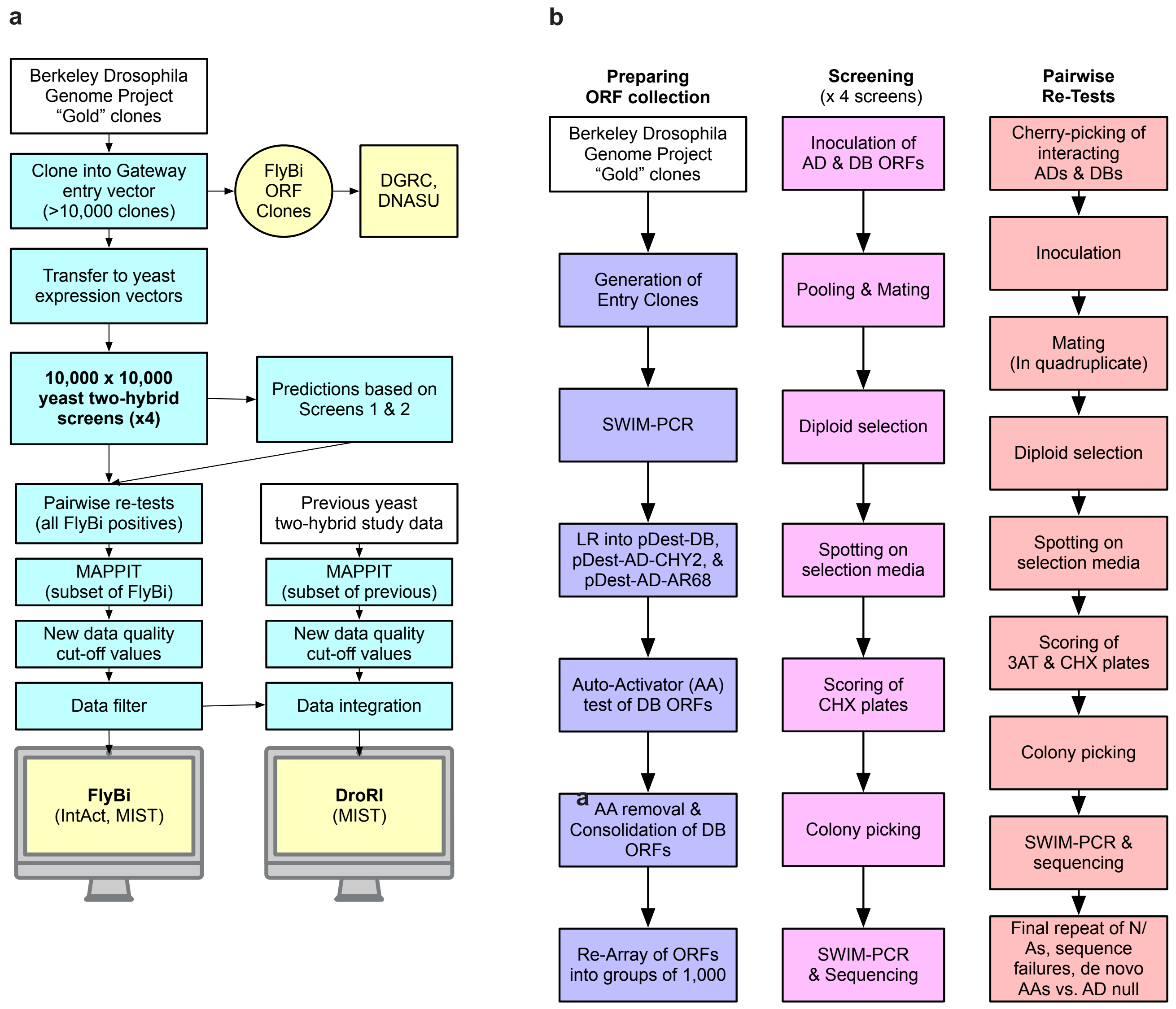

Supplementary Figure 1: FlyBi project workflow and experimental yeast two-hybrid (Y2H) approach. **a** Overall workflow. White, pre-existing physical resources or datasets. Blue, new experiments or analyses. Yellow, newly generated physical or data resources, or their locations. The project resulted in a the Y2H dataset (FlyBi) and *Drosophila* reference interactome (DroRI). **b** Y2H screen workflow. Left (purple), generation of ORF collection. Center (pink), screen pipeline. Right (orange), pairwise testing. For a detailed description of the workflow outlined in **b**, see **Supplementary Methods**. Abbreviations: SWIM-PCR, Shared-Well Interaction Mapping by sequencing; AD, activation domain; DB, DNA binding domain; ORF, open reading frame. AA, Auto-Activator; SC-Leu-Trp-His+1mM 3AT, Synthetic Complete-Leucine-Tryptophan-Histidine+ 3-amino-1,2,4-triazole; SC-Leu-His+1mM 3AT+CHX, Synthetic Complete-Leucine-Histidine+ 3-amino-1,2,4-triazole+ Cycloheximide; SC-Leu-Trp, Synthetic Complete-Leucine-Tryptophan.

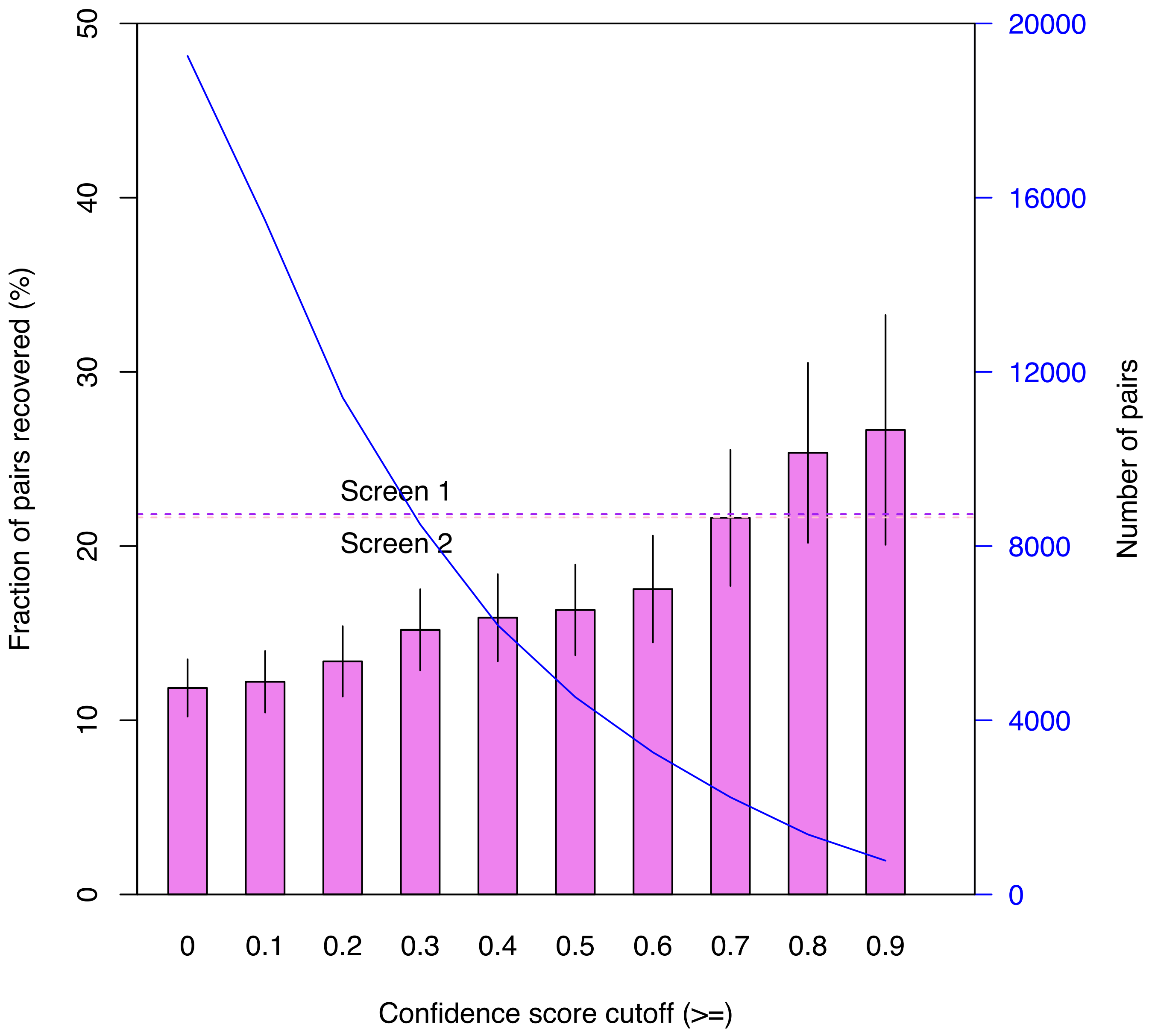

Supplemental Figure 2: Experimental evaluation of CuraGen pairs at different CuraGen confidence score cutoffs in MAPPIT assay. Blue curve, the declining number of pairs left in the dataset at the indicated CuraGen stringency cutoffs is applied across all pairs. Dashed line, the recovery rates of FlyBi screens 1 and 2 (i.e., the FlyBi screens performed in the most comparable screen version). Height of the bars indicates fraction of positives with the cutoffs (n= 371, 327, 267, 220, 198, 186, 138, 96, 59, and 41 from left to right). Error bars shown as fraction of positives ± standard error of the proportion. Source data are in **Supplementary Data 6**.

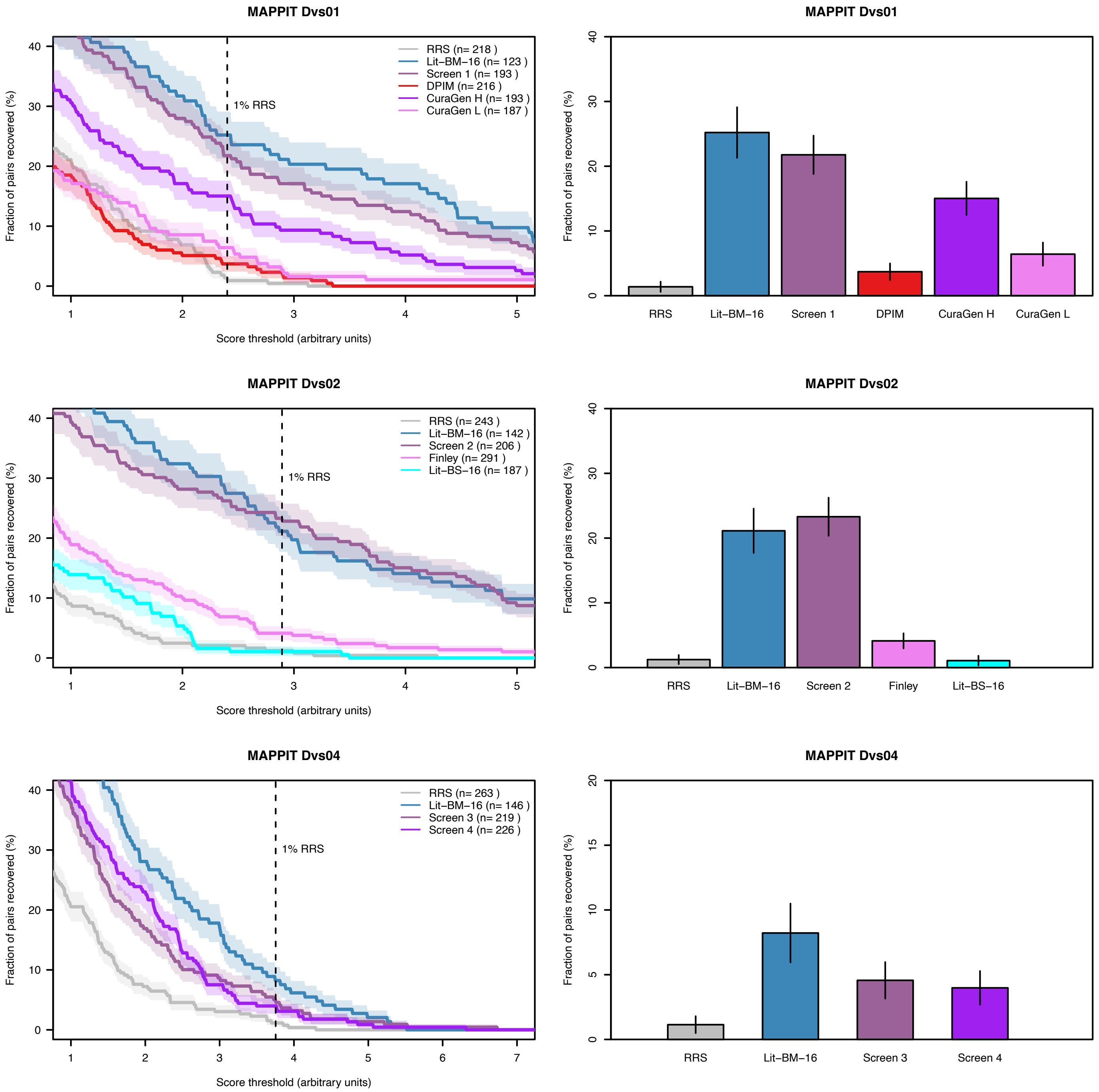

Supplementary Figure 3: Experimental validation of pairs from FlyBi and other datasets in the MAPPIT assay. The MAPPIT configurations were N-N (top and middle) or N-C (bottom), in accordance with configurations of version 1 (N-N) and 3 (N-C) in the FlyBi Y2H screens. Clouds around solid lines indicate standard error of the proportion. Left panels, titration plots showing recovery rates at different thresholds. Right panels, bar plots showing recovery rate at a cutoff value for which the random reference set (RRS) scores at a rate of 1%. Numbers of samples n for each dataset presented in plot legends. Error bars shown as fraction of positives ± standard error of the proportion. Source data are in **Supplementary Data 6**.

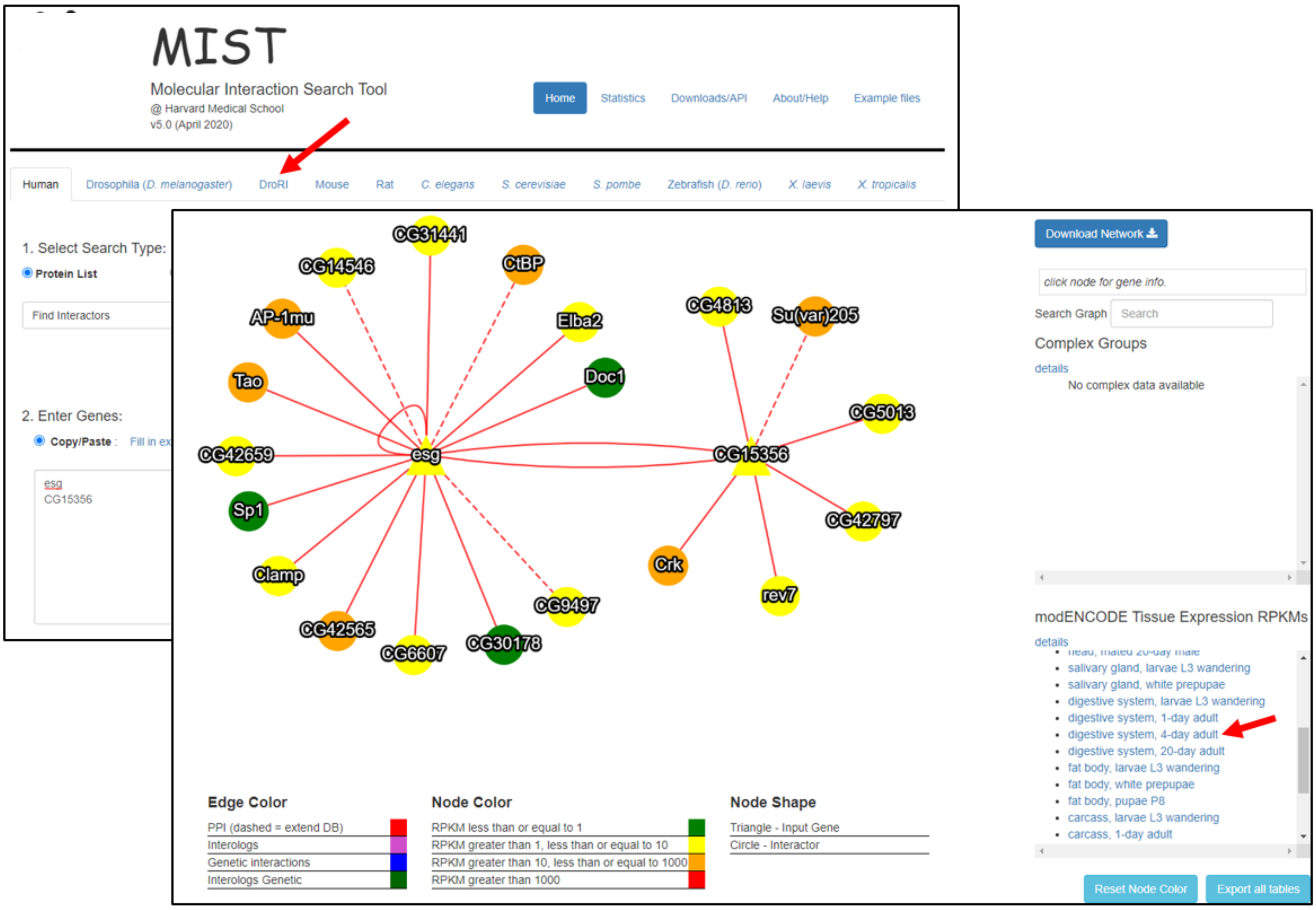

Supplementary Figure 4: Integration of DroRI network and transcriptomics data at MIST. Interactions in the DroRI master network can be queried from a dedicated tab at MIST (top left, red arrow). Users have the option to project expression levels as reported by modENCODE tissue-specific RNA-seq datasets onto the network (bottom right, red arrow). The search input is either a gene or a list of genes. An example results page for a query with *escargot* (*esg*) and *CG15356* is shown. Solid edges, binary interactions identified in the FlyBi screens; dotted edge, binary interactions curated from the literature. Node colors reflect expression levels for the selected dataset. In this example, the network is overlayed with modENCODE RNA-seq data for the digestive system of 4-day adults. Source: screenshots from <https://fgrtools.hms.harvard.edu/MIST/>.

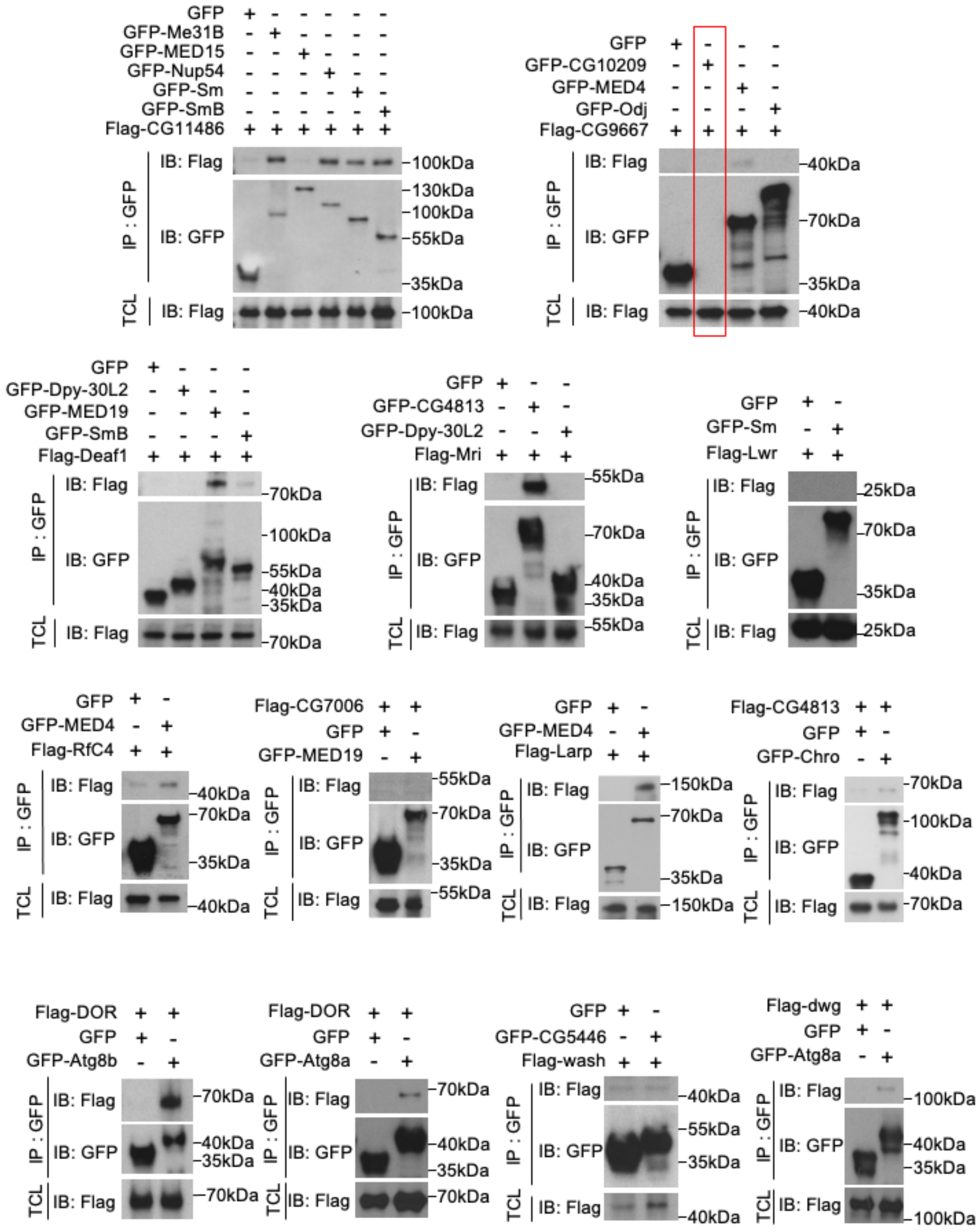

Supplementary Figure 5: Experimental validation of interactions between putative autophagy-related proteins by co-immunoprecipitation. Open reading frames (ORFs) were fused in-frame with epitope tags or GFP, co-expressed in *Drosophila* cells, and subjected to co-IP followed by detection with antibodies as indicated. See also summary of results in **Suppl. Table 2**. Experiments were repeated three times independently with similar results. Source data are provided in the **Source Data** file.

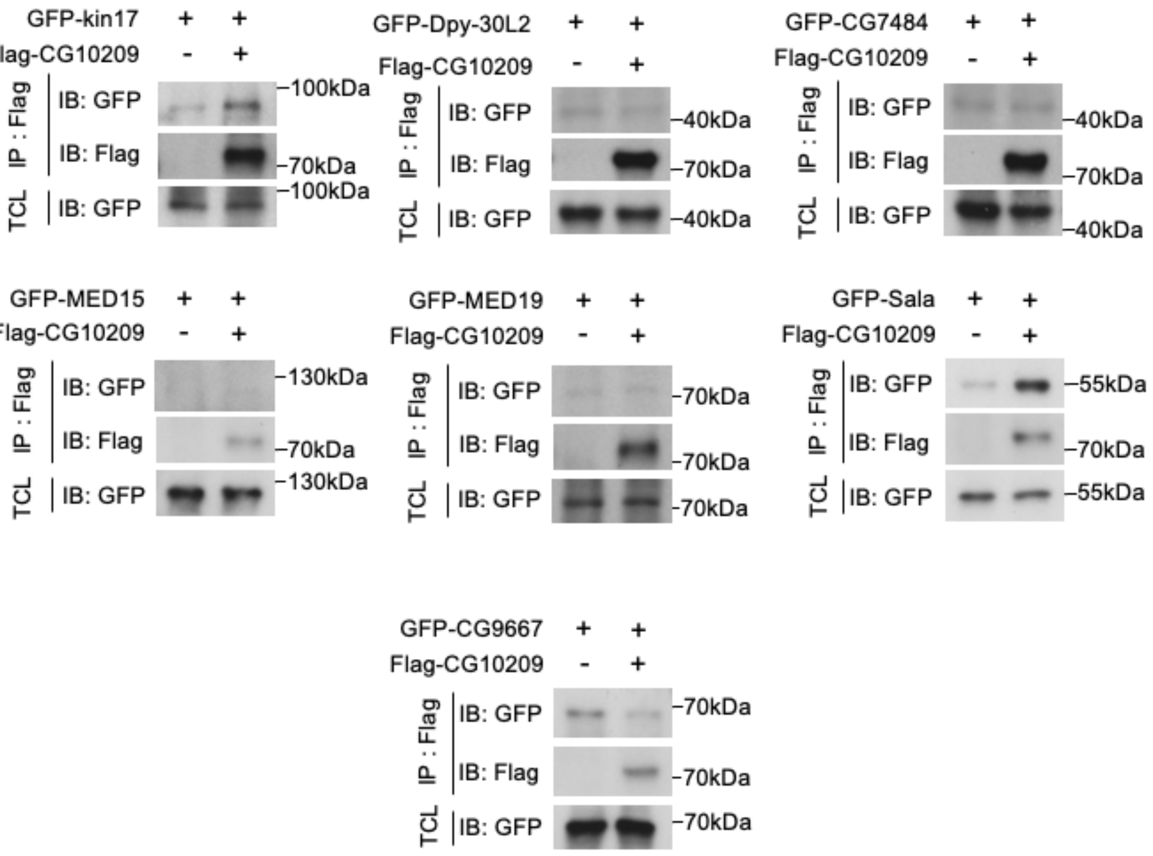

Supplementary Figure 6: Experimental validation of interactions between CG10209 and other putative autophagy-related proteins by co-immunoprecipitation (co-IP). As GFP-tagged CG10209 is undetectable (Supplemental Figure COIP-1), we designed a smaller Flag-tagged form of CG10209 and enriched it using Flag-beads for immunoprecipitation. Open reading frames (ORFs) were fused in-frame with epitope tags or GFP, co-expressed in *Drosophila* cells, and subjected to co-IP followed by detection with antibodies as indicated. See also summary of results in **Suppl. Table 2**. Experiments were repeated three times independently with similar results. Source data are provided in the **Source Data** file.

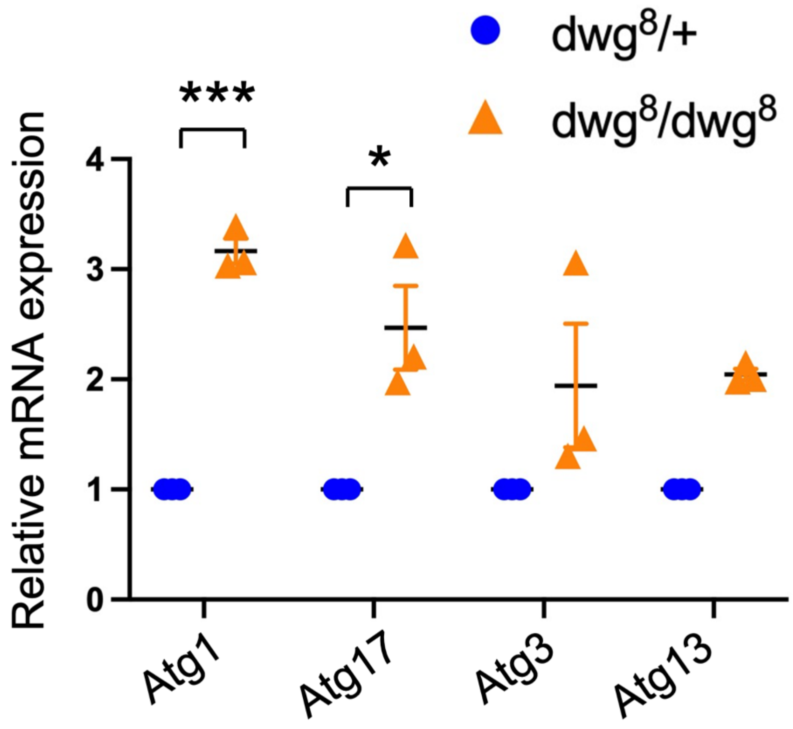

Supplementary Figure 7: Increased expression of *Atg* transcripts in *dwg* mutants. Relative mRNA expression of *Atg1, Atg17, Atg3,* and *Atg13* genes in control (*dwg*^{8}/+) and *dwg* mutants (*dwg*^{8}/*dwg*^{8}). Measurements shown are mean ± SEM. One-Way ANOVA followed by Tukey's multiple comparisons test was performed to identify significant differences; data is expressed as means ± SEM of three independent experiments; ***P<0.001 (p=0.0002); *P<0.05 (p=0.0108). Source data are provided in the **Source Data** file.

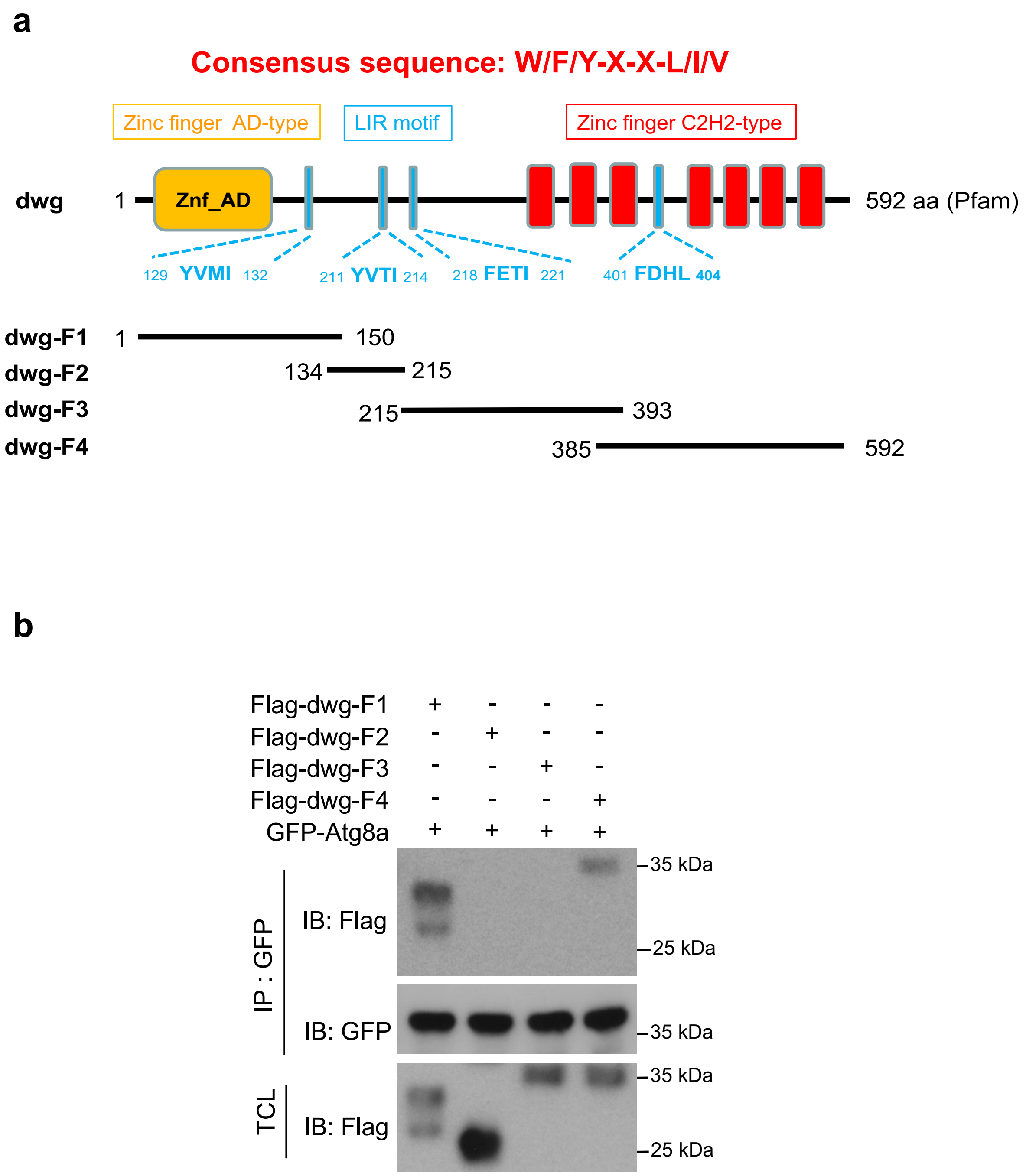

Supplementary Figure 8: Mapping the Dwg-Atg8a interaction regions. a Schematic representation of the domain structures and LIR (LC3-interacting region) motifs, and deletion mutants of *dwg*. Orange, AD-type zinc finger motif. Blue, LIR motifs. Red, C2H2-type zinc finger motifs. **b** Atg8a interacts with the first and fourth LIR motifs of dwg. S2R+ cells transfected with *GFP-Atg8a* and *Flag-dwg-F1-4* for 48 hr followed by immunoprecipitation with anti-GFP nanobody. The immunoprecipitated proteins and total cell lysates were analyzed by immunoblotting with antibodies as indicated. Experiments were repeated three times independently with similar results. Source data are provided in the **Source Data** file.

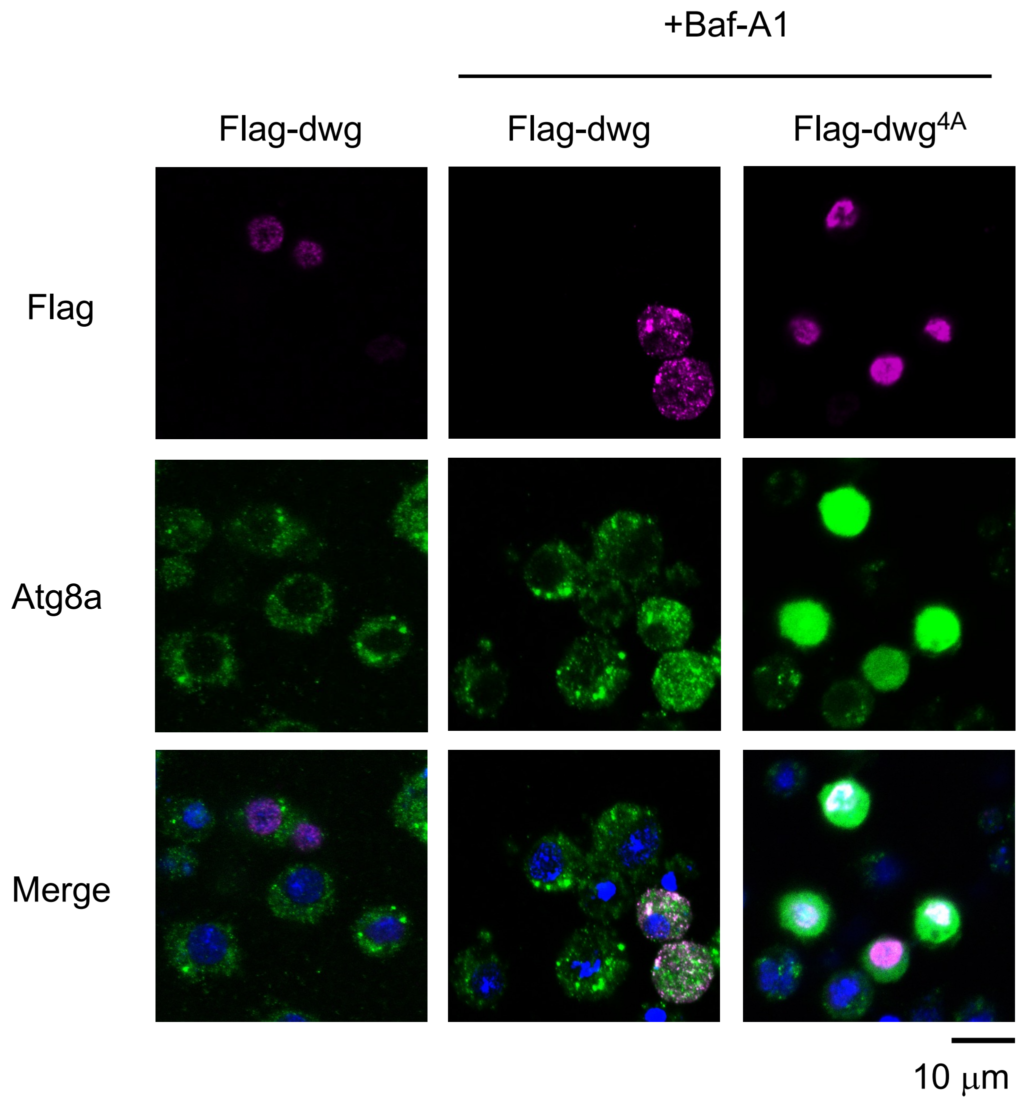

Supplementary Figure 9: Disruption of the interaction between Dwg and Atg8a inhibits autophagy. In untreated S2R+ cells, Dwg is localized to the nucleus. Bafilomycin A1 (Baf-A1) treatment inhibits autophagosome degradation and increases detectable Dwg in the cytoplasm and co-localization of Dwg with Atg8a punctae. A Dwg variant with LIR motif mutations (Dwg^{4A}) is restricted to the nucleus and strongly suppresses autophagy. Magenta, anti-Flag detection of Flag-tagged Dwg. Green, Atg8a. Blue, DAPI. Experiments were repeated three times independently with similar results. Scale bar: 10 μm. Source data are provided in the **Source Data** file.

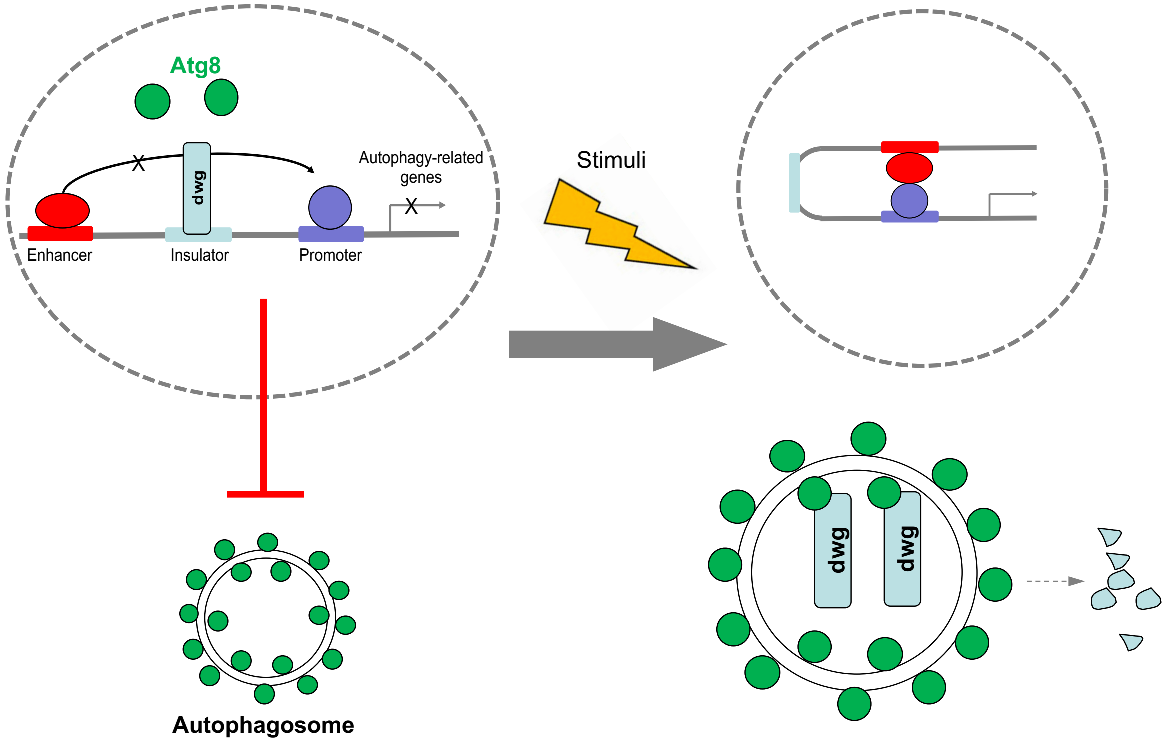

Supplementary Fig. 10: Working model of regulation of Dwg by Atg8a/autophagy. Under normal conditions, Dwg binds to insulator elements and inhibits transcription of *ATG* genes. Following a stimulus such as starvation, autophagy is induced, and Atg8a associates with and transports Dwg from the nucleus to autophagosomes for degradation. Gray line, chromosome. Red bar, enhancer sequence. Light blue bar, insulator sequence. Dark blue bar, promoter sequence. Green circles, Atg8 protein. Red oval, enhancer-binding protein. Light blue rectangle, Dwg protein. Dark blue circle, promoter-binding protein. Oval or circle with dotted line (top), nucleus. Circles with double lines (bottom), autophagosome.

2. Supplementary Tables

Supplementary Table 1: Contingency table for FlyBi and DPiM datasets.

	FlyBi interaction	FlyBi no interaction
**DPiM interaction**	72	8,133
**DPiM no interaction**	3,386	17,235,188

Note: Protein pairs were limited to the space of protein pairs tested by both assay types.

Supplementary Table 2: Results of co-immunoprecipitation in *Drosophila* cells of Flag- and GFP-tagged proteins from the putative autophagy network.

Flag-tagged protein (size[1])	GFP-tagged protein (size[1])	Interaction Detected by CoIP
CG11486 (100)	Me31B (91)	yes
CG11486	MED15 (120)	no
CG11486	Nup54 (104)	yes
CG11486	Sm (92)	yes
CG11486	SmB (61)	yes
CG9667 (45)	CG10209 (96)	no
CG9667	MED4 (68)	yes
CG9667	Odj (90)	no
Deaf1 (76)	Dpy-30L2 (50)	no
Deaf1	MED19 (75)	yes
Deaf1	SmB (61)	yes
RfC4 (52)	MED4 (68)	yes
CG7006 (36)	MED19 (75)	no
Larp (121)	MED4 (68)	yes
CG4813 (59)	Chro (141)	yes
Lwr (33)	Sm (92)	no
DOR (60)	Atg8b (54)	yes
DOR	Atg8a (54)	yes
Dwg[2] (100)	Atg8a (54)	yes
Wash (68)	CG5446 (49)	no
Mri (63)	CG4813 (84)	yes
Mri	Dpy-30L2 (50)	no
CG10209 (75)	Kin17 (95)	yes
CG10209	Dpy-30L2 (50)	no
CG10209	CG7484 (60)	no
CG10209	MED15 (120)	no
CG10209	MED19 (75)	no
CG10209	Sala (54)	yes
CG10209	CG9667 (70)	no

[1] Observed size of the fusion protein with tag, in kDa.

[2] Expected size, 81 kDa. The observed size of our Dwg fusion protein (100 kDa) is consistent with a report by [65], who similarly observed that Dwg migrates at a higher molecular weight than expected.

Supplementary Table 3: Attributes of the yeast expression vectors used in this study.

**Plasmid Vector**	**pDEST-DB (used for both assay versions)**	**pDEST-AD-CHY2 (used for assay version 1)**	**pDEST-AD-AR68 (used for assay version 3)**
**Fusion**	Gal4-DB (aa 1-147)	Gal4-AD (aa 768-881)	Gal4-AD (aa 768-881)
**Fusion location**	N-term	N-term	C-term
**Promoter**	Truncated *ADH1* promoter (-701 to +1)	Truncated *ADH1* promoter (-701 to +1)	Truncated *ADH1* promoter (-410 to +1)
**Yeast replication origin**	CEN	CEN	2 micron
**Linker**	SRSNQ	GGSNQ	VDGTA
**Terminator**	*ADH1* Term	*ADH1* Term	*ADH1* Term
**Selection marker**	AmpR	AmpR	AmpR

AD, activation domain; DB, DNA binding domain; N-term, amino-terminal fusion; C-term, carboxy-terminal fusion; CEN, centromere; *ADH1* Term, *ADH1* terminator sequence; AmpR, ampicillin resistance.

Supplementary Table 4: Primers used for SWIM-seq.

	**AD**		**DB**	
	**SWIM**	**Universal**	**SWIM**	**Universal**
**Forward**	5'-AGACGTGTGCTCTTCCGATCT NNNNNNNNNNNNNCGATGATGAA GATACCCCACCA-3’	5'-CGCGTTTGGAATCACTACAGGG-3’	5'-AGACGTGTGCTCTTCCGATCT NNNNNNNNNNNNNGGTCAAAGACA GTTGACTGTATCGT-3’	5'-GGCTTCAGTGGAGACTGATATGCCTC-3’
**Reverse**	5'-GGAGACTTGACCAAACCTCTGGCG-3’	5'-GGAGACTTGACCAAACCTCTGGCG-3’	5'-GGAGACTTGACCAAACCTCTGGCG-3’	5'-GGAGACTTGACCAAACCTCTGGCG-3’

AD, activation domain; DB, DNA binding domain.

“N”s denote the 13-mer well index.

3. Supplementary Methods

The following detailed legend for Supplementary Fig. 1b provides details regarding the yeast two-hybrid (Y2H) workflow: Suppl. Fig. 1b, left (purple), generation of the ORF collection. ORFs were amplified with M13 SWIM primers and successfully sequenced ORFs were transferred into yeast expression vectors via Gateway cloning and transformed into yeast (pDEST-DB into Y8930, pDEST-AD-*CYH2* and pDEST-AD-AR68 into Y8800). ORFs in pDEST-DB were tested for auto-activation by spotting on Synthetic Complete media lacking Leucine and Histidine (SC-Leu-His) plates. Strong auto-activators (AA) were removed and the yeast ORFs were re-arrayed into groups of 1,000. Suppl. Fig. 1b, center (pink), screening pipeline. AD and DB ORFs were inoculated in corresponding selection media and incubated at 30˚C overnight. The next day, 1,000 AD-ORFs were pooled to obtain a kilo-pool and mated against a single DB in YPD media. After overnight incubation at 30˚C, 10 µl of the mated yeast culture was transferred into Synthetic Complete media lacking Leucine and Tryptophan (SC-Leu-Trp media) to select for diploid yeast cells. The following day, diploid yeast cells were spotted on SC-Leu-Trp-His+1mM 3AT selection media and SC-Leu-His+1mM 3AT+CHX. Colonies growing on SC-Leu-Trp-His+1mM 3AT plates but not on SC-Leu-His+1mM 3AT+CHX plates, were picked up to 3 times from each spot. Lysates, SWIM PCR and sequencing were performed to identify interacting pairs. Suppl. Fig. 1b, right (orange), pairwise testing. Interacting pairs were arrayed into 96-well plates for AD and DB respectively, inoculated into selection media and after incubation overnight at 30˚C mated in quadruplicates. The next day, diploid selection was performed by transferring 10 µl mated yeast culture into SC-Leu-Trp. After growth overnight at 30˚C, diploid yeast cells were spotted on selection media. For positive interactions growing on 3AT plates only and not on selection plates containing CHX, one out of four replicates was picked, SWIM PCR performed and sequence confirmed. A final pairwise test was performed for pairs that scored as de novo auto-activators or NAs (not applicable due to invalid scores) in the pairwise test. For those pairs, each DB-ORF was separately mated with an AD-null plasmid (no ORF in the cloning site) in parallel with the AD interactor. If a yeast colony grew more strongly when mated to the corresponding AD-

ORF than the AD-null strain, the PPI was considered valid and added to the dataset after sequence confirmation. Abbreviations: SWIM-PCR, Shared-Well Interaction Mapping by sequencing; AD, activator domain; DB, DNA binding domain; ORF, open reading frame; AA, Auto-Activator; SC-Leu-Trp-His+1mM 3AT Synthetic Complete-Leucine-Tryptophan-Histidine+ 3-amino-1,2,4-triazole; SC-Leu-His+1mM 3AT+CHX Synthetic Complete-Leucine-Histidine+ 3-amino-1,2,4-triazole+ Cycloheximide; SC-Leu-Trp, Synthetic Complete-Leucine-Tryptophan; YPD media, yeast extract peptone dextrose media.

4. Supplementary Notes

Below, we provide detailed statistical information related to Fig. 2a, 2c, and 2d-f.

Figure 2a, shown are means ± standard errors of the means (SEM). Specific values are provided below.

Protein-Protein Interactions (PPIs)

terms	mean	SEM
PPI fly only	52.48057	0.948614
PPI interolog	16.80495	0.178605

Genetic Interactions (GIs)

terms	mean	SEM
GI fly only	15.71534	0.252687
GI yeast similar partner	14.49481	0.301535

Figure 2c, shown are means ± standard errors of the means (SEM). Specific values are provided below.

FlyBi

terms	mean	SEM
BP	9.847150922	0.11847
CC	29.05725	0.556465
MF	14.91105281	0.284115
Phenotype	4.2139	0.055826
MIST complex all	21.82448779	0.444919
MIST complex only indirect	5.003812163	0.109735
COMPLEATDB	17.31634279	0.331743

Lit-BM

terms	mean	SEM
BP	37.95981429	0.658654
CC	56.84576667	0.710396
MF	27.95158333	0.294271
Phenotype	17.58085714	0.311373

MIST complex all	180.587403	2.220773
MIST complex only indirect	4.583720654	0.063407
COMPLEATDB	46.73468111	0.619935

Figure 2d, empirical $p < 0.001$ for both FlyBi data and Lit-BM. Figure 2E, empirical $p < 0.001$ for both FlyBi data and Lit-BM. Figure 2F, empirical $p = 0.011$ for FlyBi data and $p < 0.001$ for Lit-BM. Specific values are provided below.

**Figure panel and dataset**	**Real value**	**Mean of randoms**
D-FlyBi	71.09599	55.69748
D-Lit-BM	79.48718	71.75689
E-FlyBi	491	192.123
E-Lit-BM	2094	319.394
F-FlyBi	4.983969	4.387867
F-Lit-BM	5.713529	4.687895
