# Supplementary material for: Next-generation large-scale binary protein interaction network for Drosophila melanogaster
Source: Nat Commun. 2023 Apr 15;14:2162. doi: 10.1038/s41467-023-37876-0 (PMC10105736; doi:10.1038/s41467-023-37876-0)
Supplement: Supplementary file 3 — Description of Additional Supplementary Files [file 41467_2023_37876_MOESM3_ESM.pdf]

Description of Additional Supplementary Files

File Name: Supplementary Data 1
Description: List of *Drosophila* protein pairs that comprise the positive reference set (PRS) or the random reference set (RRS).

File Name: Supplementary Data 2
Description: Lit-BM-16, a list of protein pairs in the literature for which multiple lines of evidence suggest a binary interaction (built in 2017 based on annotations available through 2016).

File Name: Supplementary Data 3
Description: Lit-BM-20, a list of protein pairs in the literature for which multiple lines of evidence suggest a binary interaction (built in 2021 based on annotations available through 2020).

File Name: Supplementary Data 4
Description: Lit-BS, a list of protein pairs in the literature for which only one line of evidence suggests a binary interaction.

File Name: Supplementary Data 5
Description: FlyBi dataset, the full list of protein pairs that were positive in one or more of the four all-by-all yeast two-hybrid screens described in this study.

File Name: Supplementary Data 6
Description: Results of MAmmalian Protein-Protein Interaction Trap (MAPPIT) analysis.

File Name: Supplementary Data 7
Description: Lists of interactors with known autophagy components (list 1) and extensions of that list based on interactions in the FlyBi dataset (lists 2 and 3).

File Name: Supplementary Data 8
Description: Results of RNAi screens for autophagy-related phenotypes for the putative autophagy network (sheet 1) or a random set (sheet 2). Columns D and E report phenotypes observed in the *Atg1* over-expression modifier assay in the adult eye. Columns F and G report phenotypes observed in the mCherry-Atg8 distribution assay in the larval fat body. NIG, National Institute of Genetics of Japan collection RNAi fly stocks. TRiP, Transgenic RNAi Project collection RNAi fly stocks. VDRC, Vienna Drosophila Research Center collection RNAi fly stocks.

File Name: Supplementary Data 9
Description: Sequences of primers used to PCR amplify open reading frames (ORFs) from Berkeley *Drosophila* Genome Project clone resource for production of the FlyBi ORF clone resource.
