# Supplementary material for: Next-generation large-scale binary protein interaction network for Drosophila melanogaster
Source: Nat Commun. 2023 Apr 15;14:2162. doi: 10.1038/s41467-023-37876-0 (PMC10105736; doi:10.1038/s41467-023-37876-0)
Supplement: Supplementary file 13 — Reporting Summary [file 41467_2023_37876_MOESM13_ESM.pdf]

nature portfolio

Corresponding author(s): Mohr, Perrimon, Vidal, Celniker

Last updated by author(s): Feb 25, 2023

Reporting Summary

Nature Portfolio wishes to improve the reproducibility of the work that we publish. This form provides structure for consistency and transparency in reporting. For further information on Nature Portfolio policies, see our Editorial Policies and the Editorial Policy Checklist.

Statistics

For all statistical analyses, confirm that the following items are present in the figure legend, table legend, main text, or Methods section.

n/a	Confirmed	
☐	☒	The exact sample size (*n*) for each experimental group/condition, given as a discrete number and unit of measurement
☒	☐	A statement on whether measurements were taken from distinct samples or whether the same sample was measured repeatedly
☐	☒	The statistical test(s) used AND whether they are one- or two-sided<br>*Only common tests should be described solely by name; describe more complex techniques in the Methods section.*
☒	☐	A description of all covariates tested
☐	☒	A description of any assumptions or corrections, such as tests of normality and adjustment for multiple comparisons
☐	☒	A full description of the statistical parameters including central tendency (e.g. means) or other basic estimates (e.g. regression coefficient) AND variation (e.g. standard deviation) or associated estimates of uncertainty (e.g. confidence intervals)
☐	☒	For null hypothesis testing, the test statistic (e.g. *F*, *t*, *r*) with confidence intervals, effect sizes, degrees of freedom and *P* value noted<br>*Give P values as exact values whenever suitable.*
☒	☐	For Bayesian analysis, information on the choice of priors and Markov chain Monte Carlo settings
☐	☒	For hierarchical and complex designs, identification of the appropriate level for tests and full reporting of outcomes
☒	☐	Estimates of effect sizes (e.g. Cohen's *d*, Pearson's *r*), indicating how they were calculated

Our web collection on statistics for biologists contains articles on many of the points above.

Software and code

Policy information about availability of computer code

Data collection

Code relevant to Y2H data was as published in Luck et al. 2020 and available at <https://github.com/CCSB-DFCI/HuRI_paper>

Data analysis

For ChIP-seq analysis, low-quality reads and adaptor primer sequences were trimmed using Trim Galore 0.6.4 (https://github.com/FelixKrueger/TrimGalore). Trimmed reads were mapped using bowtie2 2.3.5.1 with the additional argument "-q --local"84. Samtools 1.6 was used to sort, filter unique reads, and convert file format to bam files. Peak calling was performed with MACS2 2.2.6 with additional parameter "-B --SPMR -f BAMPE -g dm". Peaks were annotated with HOMER 4.1187. DeepTools 3.4.0 were used for normalizing read counts to CPM and convert bam files to bigWig format. The code for node shuffling used to generate random networks based on the FlyBi network is available at <https://github.com/moontreegy/flybi-network-analysis>. All other code is as previously published. L3 prediction code files was published in Kovacs et al. (2019) and is available at <https://doi.org/10.5281/zenodo.2008592>. Code relevant to Y2H and PPI analyses was published in Luck et al. 2020 and is available at <https://github.com/CCSB-DFCI/HuRI_paper>. SAFE analysis software version 1.5 was used in this study; SAFE was published by Baryshnikova et al. (2016) and is available at https://github.com/baryshnikova-lab/safepy. FBgn IDs were updated and validated using <http://flybase.org/convert/id>. Band sizes were calculated using BioRad Quantity One software (version 4.6.9). PCR primers for ORF amplification were designed using Primer3 release 0.9 69 (the resulting primer sequences are reported in Suppl. File 9).

For manuscripts utilizing custom algorithms or software that are central to the research but not yet described in published literature, software must be made available to editors and reviewers. We strongly encourage code deposition in a community repository (e.g. GitHub). See the Nature Portfolio guidelines for submitting code & software for further information.

Data

Policy information about availability of data

All manuscripts must include a data availability statement. This statement should provide the following information, where applicable:

- Accession codes, unique identifiers, or web links for publicly available datasets
- A description of any restrictions on data availability
- For clinical datasets or third party data, please ensure that the statement adheres to our policy

FlyBi binary interaction data are as provided in Supplementary file 5 and are also available as a table and as a downloadable data file at the FlyBi project webpage ([http://flybi.hms.harvard.edu/]). These data were also integrated with other datasets at IntAct ([https://www.ebi.ac.uk/intact/]) and in the Molecular Interaction Search Tool (MIST; [https://fgrtools.hms.harvard.edu/MIST/]). MAPPIT data is provided as Suppl. File 6. RNAi data for the autophagy-related network is provided as as Suppl. File 8. Plasmid clones and information are available from at the Drosophila Genomics Resource Center (University of Indiana, Bloomington, IN) and DNASU plasmid repository (Arizona State University, Phoenix, AZ). ORFs in the Gateway donor vector were end-read sequenced and this sequence data is available at GenBank and at the FlyBi project website (see "Genbank Accession" columns on the table at [https://flybi.hms.harvard.edu/results.php]). For a subset of 954 ORFs, the end-reads sequence spanned the full ORF; this sequence data is available at the NCBI (Project Accession ID PRJNA349744) and a list of these ORFs, along with NCBI IDs, is available at the FlyBi project website (see [https://flybi.hms.harvard.edu/clones.php]). Interaction data was deposited at EBI IntAct (<https://www.ebi.ac.uk/intact/home>) and our integrated Drosophila reference interaction (DroRI) PPIs are available at MIST (<https://fgrtools.hms.harvard.edu/MIST/>). ChIPseq data is available at NCBI GEO (Accession ID GSE220887).

Human research participants

Policy information about studies involving human research participants and Sex and Gender in Research.

Reporting on sex and gender	There were no human research participants in this study
Population characteristics	Not applicable to this study
Recruitment	Not applicable to this study
Ethics oversight	Not applicable to this study

Note that full information on the approval of the study protocol must also be provided in the manuscript.

Field-specific reporting

Please select the one below that is the best fit for your research. If you are not sure, read the appropriate sections before making your selection.

☒ Life sciences ☐ Behavioural & social sciences ☐ Ecological, evolutionary & environmental sciences

For a reference copy of the document with all sections, see nature.com/documents/nr-reporting-summary-flat.pdf

Life sciences study design

All studies must disclose on these points even when the disclosure is negative.

Sample size	Sample size was determined by the number of unique available Drosophila open reading frame (ORF) clones (yeast two-hybrid screen) or by the results of the screen, with filters applied (e.g., Y2H-detected PPIs with specific candidates) as described in the manuscript
Data exclusions	No data were excluded.
Replication	The large-scale screen was performed two times in each of two different formats (four screens in total). Follow-up studies followed standard guidelines for study design for Drosophila or other studies, e.g., three replicates for in vivo studies, and all replicates of these studies gave comparable results.
Randomization	For the yeast two-hybrid screens, all available ORFs were included (no non-random sub-sets). Subsets of results of the large-scale screen were chosen at random for MAPPIT analysis. For the in vivo autophagy study, we used known autophagy components as the start-point to build a putative autophagy network comprised of known autophagy components (list 1), interactors with those proteins (list 2) and interactors with list 2 proteins (list 3)(see Supplemental data file 7), then tested all components in the Atg1 autophagy-related assay. Positives in that assay were included in the fat body autophagy-related assay.
Blinding	Y2H screening used pooled approaches and lab automation; researchers were blind to identify of specific ORF clones in yeast until the endpoint of the assay (sequencing) revealed identity. For the genetic screens, fly stocks were labeled and data collected based on RNAi stock IDs; researchers blind to the gene targets during the assay and data collection; identities of gene targets were only revealed when look-up tables were used to associate a stock ID with a gene target.

Reporting for specific materials, systems and methods

We require information from authors about some types of materials, experimental systems and methods used in many studies. Here, indicate whether each material, system or method listed is relevant to your study. If you are not sure if a list item applies to your research, read the appropriate section before selecting a response.

Materials & experimental systems

n/a	Involved in the study
☐	☒ Antibodies
☐	☒ Eukaryotic cell lines
☒	☐ Palaeontology and archaeology
☐	☒ Animals and other organisms
☒	☐ Clinical data
☒	☐ Dual use research of concern

Methods

n/a	Involved in the study
☐	☒ ChIP-seq
☒	☐ Flow cytometry
☒	☐ MRI-based neuroimaging

Antibodies

Antibodies used

Antibodies were used for immunofluorescence (IF) or immunoblotting (WB) at the following dilutions. Rabbit polyclonal anti-GFP (A-6455, Molecular Probes), dilution factor 1:5,000 (WB); rabbit monoclonal anti-Atg8 (ab109364, Abcam), dilution factor 1:2,000 (WB) or 1:100 (IF); mouse monoclonal anti-Flag (F3165, Sigma), dilution factor 1:5,000 (WB) or 1:1,000 (IF); mouse monoclonal anti-HA (901514, Biolegend), dilution factor 1:1,000 (IF); rabbit polyclonal anti-GAPDH (GTX100118, GeneTax), dilution factor 1:10,000 (WB); goat anti-Mouse IgG (H+L) secondary antibody, Alexa Fluor 633 (A-21052, Invitrogen), dilution factor 1:1,000 (IF); donkey anti-Mouse IgG (H+L) Secondary Antibody, Alexa Fluor 555 (A-31570, Invitrogen), dilution factor 1:1,000 (IF); rabbit Anti-Mouse IgG (Light Chain Specific) (D3V2A) mAb (HRP Conjugate) (58802, Cell Signaling), dilution factor 1:1000 (WB); mouse Anti-Rabbit IgG (Light-Chain Specific) (D4W3E) mAb (HRP Conjugate) (93702, Cell Signaling), dilution factor 1:1000 (WB). For immunoprecipitation, we used a GFP Nanobody/VHH coupled to agarose beads (ChromoTek GFP-Trap Agarose, AB_2631357). For ChIP-seq, we used anti-Flag (Sigma, F3165) and the IgG antibody beads as included in SimpleChIP Plus Enzymatic Chromatin IP Kit (Cell Signaling Technology, 9005).

Validation

These are standard commercial antibodies, against well-characterized conserved protein targets or epitopes, validated by the manufacturer and reported in publications, as noted at manufacturer websites. Please see below.
1. Rabbit polyclonal anti-GFP, validated by manufacturer (https://www.thermofisher.com/antibody/product/A-6455.html?gclid=CjwKCAiArY2fBhB9EiwAWqHK6ow9ESZoZny1Nlhy2JBYrc7TiagTpf3xOzOWdGYagFy4I3Taii3xoRoCIEoQAvD_BwE&ef_id=CjwKCAiArY2fBhB9EiwAWqHK6ow9ESZoZny1Nlhy2JBYrc7TiagTpf3xOzOWdGYagFy4I3Taii3xoRoCIEoQAvD_BwE:G:s&s_kwcid=AL!3652!3!459736943987!!!g!!!10950825775!106531320406&cid=bid_pca_aup_r01_co_cp1359_pjt0000_bid00000_0se_gaw_dy_pur_con)
2. Rabbit monoclonal anti-Atg8, validated by manufacturer (https://www.abcam.com/nav/primary-antibodies/rabbit-monoclonal-antibodies/gabarapgabarapl1gabarapl2-antibody-epr4805-ab109364.html) (note, includes reports for Drosophila Atg8)
3. mouse monoclonal anti-Flag, validated by manufacturer (https://www.sigmaaldrich.com/SG/en/product/sigma/f3165)
4. mouse monoclonal anti-HA, validated by manufacturer (https://www.biolegend.com/en-us/products/anti-ha-11-epitope-tag-antibody-11071)
5. rabbit polyclonal anti-GAPDH, validated by manufacturer (https://www.genetex.com/Product/Detail/GAPDH-antibody/GTX100118)
6. Goat anti-Mouse IgG (H+L) Secondary Antibody, Alexa Fluor® 633, validated by manufacturer (https://www.thermofisher.com/antibody/product/A-21052.html?CID=AFLLO-A-21052)
7. Donkey anti-Mouse IgG (H+L) Secondary Antibody, Alexa Fluor™ 555, validated by manufacturer (https://www.thermofisher.com/antibody/product/Donkey-anti-Mouse-IgG-H-L-Highly-Cross-Adsorbed-Secondary-Antibody-Polyclonal/A-31570)
8. Rabbit Anti-Mouse IgG (Light Chain Specific) (D3V2A) mAb (HRP Conjugate), validated by manufacturer (https://www.cellsignal.com/products/secondary-antibodies/rabbit-anti-mouse-igg-light-chain-specific-d3v2a-mab-hrp-conjugate/58802)
9. Mouse Anti-Rabbit IgG (Light-Chain Specific) (D4W3E) mAb (HRP Conjugate), validated by manufacturer (https://www.cellsignal.com/products/secondary-antibodies/mouse-anti-rabbit-igg-light-chain-specific-d4w3e-mab-hrp-conjugate/93702)

Eukaryotic cell lines

Policy information about cell lines and Sex and Gender in Research

Cell line source(s)

The S. cerevisiae (yeast) used in this study were lab strains derivative of S288C and the genotypes are described in detail in the manuscript. The mammalian cell line used in this study was a Tavernier lab copy of HEK293T.

Authentication

Authentication of HEK293T was performed by genome sequencing as described in Lin et al. (2014) PMCID: PMC4166678

Mycoplasma contamination

Standard methods for monitoring for mycoplasma were applied; no contamination was detected.

Commonly misidentified lines
(See ICLAC register)

"HEK," but not the "HEK293T" cell line we used, appears on the list of commonly misidentified lines. We authenticated the cell line as indicated above and further note that the identity of the cell line should have little or no impact on the findings reported, given our use of the cells, i.e., for MAPPIT assays with Drosophila proteins

Animals and other research organisms

Policy information about studies involving animals; ARRIVE guidelines recommended for reporting animal research, and Sex and Gender in Research

Laboratory animals	Drosophila melanogaster lab strains
Wild animals	This study did not involve wild animals
Reporting on sex	Only male Drosophila were scored for the Atg1 eye phenotype to avoid differences in eye size due to sex. For the larval fat body assay, Drosophila larvae of both sexes were included.
Field-collected samples	This study did not involve field-collected samples
Ethics oversight	No ethical approval or guidance was required because the lab animal component of this study was limited to Drosophila melanogaster

Note that full information on the approval of the study protocol must also be provided in the manuscript.

ChIP-seq

Data deposition

☒ Confirm that both raw and final processed data have been deposited in a public database such as GEO.

☒ Confirm that you have deposited or provided access to graph files (e.g. BED files) for the called peaks.

Data access links<br>*May remain private before publication.*	https://www.ncbi.nlm.nih.gov/geo/query/acc.cgi?acc=GSE220887
Files in database submission	LIB045499_CHS00173889_S1_L001_R1.fastq.bz2 , LIB045499_CHS00173889_S1_L001_R2.fastq.bz2, LIB045499_CHS00173889_S1_L002_R1.fastq.bz2, LIB045499_CHS00173889_S1_L002_R2.fastq.bz2, LIB045499_CHS00173889_S1_L003_R1.fastq.bz2, LIB045499_CHS00173889_S1_L003_R2.fastq.bz2, LIB045499_CHS00173889_S1_L004_R1.fastq.bz2, LIB045499_CHS00173889_S1_L004_R2.fastq.bz2, LIB045499_CHS00173890_S2_L001_R1.fastq.bz2, LIB045499_CHS00173890_S2_L001_R2.fastq.bz2, LIB045499_CHS00173890_S2_L002_R1.fastq.bz2, LIB045499_CHS00173890_S2_L002_R2.fastq.bz2, LIB045499_CHS00173890_S2_L003_R1.fastq.bz2, LIB045499_CHS00173890_S2_L003_R2.fastq.bz2, LIB045499_CHS00173890_S2_L004_R1.fastq.bz2, LIB045499_CHS00173890_S2_L004_R2.fastq.bz2, LIB045499_CHS00173892_S4_L001_R1.fastq.bz2, LIB045499_CHS00173892_S4_L001_R2.fastq.bz2, LIB045499_CHS00173892_S4_L002_R1.fastq.bz2, LIB045499_CHS00173892_S4_L002_R2.fastq.bz2, LIB045499_CHS00173892_S4_L003_R1.fastq.bz2, LIB045499_CHS00173892_S4_L003_R2.fastq.bz2, LIB045499_CHS00173892_S4_L004_R1.fastq.bz2, LIB045499_CHS00173892_S4_L004_R2.fastq.bz2, LIB045499_CHS00173893_S5_L001_R1.fastq.bz2, LIB045499_CHS00173893_S5_L001_R2.fastq.bz2, LIB045499_CHS00173893_S5_L002_R1.fastq.bz2, LIB045499_CHS00173893_S5_L002_R2.fastq.bz2, LIB045499_CHS00173893_S5_L003_R1.fastq.bz2, LIB045499_CHS00173893_S5_L003_R2.fastq.bz2, LIB045499_CHS00173893_S5_L004_R1.fastq.bz2, LIB045499_CHS00173893_S5_L004_R2.fastq.bz2, LIB045499_CHS00173895_S7_L001_R1.fastq.bz2, LIB045499_CHS00173895_S7_L001_R2.fastq.bz2, LIB045499_CHS00173895_S7_L002_R1.fastq.bz2, LIB045499_CHS00173895_S7_L002_R2.fastq.bz2, LIB045499_CHS00173895_S7_L003_R1.fastq.bz2, LIB045499_CHS00173895_S7_L003_R2.fastq.bz2, LIB045499_CHS00173895_S7_L004_R1.fastq.bz2, LIB045499_CHS00173895_S7_L004_R2.fastq.bz2, LIB045499_CHS00173896_S8_L001_R1.fastq.bz2, LIB045499_CHS00173896_S8_L001_R2.fastq.bz2, LIB045499_CHS00173896_S8_L002_R1.fastq.bz2, LIB045499_CHS00173896_S8_L002_R2.fastq.bz2, LIB045499_CHS00173896_S8_L003_R1.fastq.bz2, LIB045499_CHS00173896_S8_L003_R2.fastq.bz2, LIB045499_CHS00173896_S8_L004_R1.fastq.bz2, LIB045499_CHS00173896_S8_L004_R2.fastq.bz2, dwg_vs_control_summits.bed, dwg_vs_input_summits.bed
Genome browser session<br>(e.g. UCSC)	No longer applicable.

Methodology

Replicates	Two biological replicates for each condition (2 input, 2 IgG control, 2 IP-Flag-Dwg).
Sequencing depth	Sequencing Depth: LIB045499_CHS00173889_S1_L001_R1, 1.8; LIB045499_CHS00173889_S1_L001_R2, 1.8; LIB045499_CHS00173889_S1_L002_R1, 1.8; LIB045499_CHS00173889_S1_L002_R2, 1.8; LIB045499_CHS00173889_S1_L003_R1, 1.9; LIB045499_CHS00173889_S1_L003_R2, 1.9; LIB045499_CHS00173889_S1_L004_R1, 1.8; LIB045499_CHS00173889_S1_L004_R2, 1.8; LIB045499_CHS00173890_S2_L001_R1, 3; LIB045499_CHS00173890_S2_L001_R2, 3; LIB045499_CHS00173890_S2_L002_R1, 2.9; LIB045499_CHS00173890_S2_L002_R2, 2.9; LIB045499_CHS00173890_S2_L003_R1, 3; LIB045499_CHS00173890_S2_L003_R2, 3; LIB045499_CHS00173890_S2_L004_R1, 2.9; LIB045499_CHS00173890_S2_L004_R2, 2.9; LIB045499_CHS00173891_S3_L001_R1, 2.4; LIB045499_CHS00173891_S3_L001_R2, 2.4; LIB045499_CHS00173891_S3_L002_R1, 2.3; LIB045499_CHS00173891_S3_L002_R2, 2.3; LIB045499_CHS00173891_S3_L003_R1, 2.4; LIB045499_CHS00173891_S3_L003_R2, 2.4; LIB045499_CHS00173891_S3_L004_R1, 2.3; LIB045499_CHS00173891_S3_L004_R2,

2.3; LIB045499_CHS00173892_S4_L001_R1, 3.8; LIB045499_CHS00173892_S4_L001_R2, 3.8; LIB045499_CHS00173892_S4_L002_R1, 3.8; LIB045499_CHS00173892_S4_L002_R2, 3.8; LIB045499_CHS00173892_S4_L003_R1, 3.9; LIB045499_CHS00173892_S4_L003_R2, 3.9; LIB045499_CHS00173892_S4_L004_R1, 3.8; LIB045499_CHS00173892_S4_L004_R2, 3.8; LIB045499_CHS00173893_S5_L001_R1, 3; LIB045499_CHS00173893_S5_L001_R2, 3; LIB045499_CHS00173893_S5_L002_R1, 2.9; LIB045499_CHS00173893_S5_L002_R2, 2.9; LIB045499_CHS00173893_S5_L003_R1, 3; LIB045499_CHS00173893_S5_L003_R2, 3; LIB045499_CHS00173893_S5_L004_R1, 2.9; LIB045499_CHS00173893_S5_L004_R2, 2.9; LIB045499_CHS00173894_S6_L001_R1, 3.4; LIB045499_CHS00173894_S6_L001_R2, 3.4; LIB045499_CHS00173894_S6_L002_R1, 3.3; LIB045499_CHS00173894_S6_L002_R2, 3.3; LIB045499_CHS00173894_S6_L003_R1, 3.4; LIB045499_CHS00173894_S6_L003_R2, 3.4; LIB045499_CHS00173894_S6_L004_R1, 3.3; LIB045499_CHS00173894_S6_L004_R2, 3.3; LIB045499_CHS00173895_S7_L001_R1, 4.4; LIB045499_CHS00173895_S7_L001_R2, 4.4; LIB045499_CHS00173895_S7_L002_R1, 4.3; LIB045499_CHS00173895_S7_L002_R2, 4.3; LIB045499_CHS00173895_S7_L003_R1, 4.5; LIB045499_CHS00173895_S7_L003_R2, 4.5; LIB045499_CHS00173895_S7_L004_R1, 4.4; LIB045499_CHS00173895_S7_L004_R2, 4.4; LIB045499_CHS00173896_S8_L001_R1, 5.8; LIB045499_CHS00173896_S8_L001_R2, 5.8; LIB045499_CHS00173896_S8_L002_R1, 5.7; LIB045499_CHS00173896_S8_L002_R2, 5.7; LIB045499_CHS00173896_S8_L003_R1, 5.9; LIB045499_CHS00173896_S8_L003_R2, 5.9; LIB045499_CHS00173896_S8_L004_R1, 5.7; LIB045499_CHS00173896_S8_L004_R2, 5.7; Number of uniquely mapped reads: LIB045499_CHS00173889_S1_L001, 1236478; LIB045499_CHS00173889_S1_L002, 1226998; LIB045499_CHS00173889_S1_L003, 1270909; LIB045499_CHS00173889_S1_L004, 1230250; LIB045499_CHS00173890_S2_L001, 2086760; LIB045499_CHS00173890_S2_L002, 2061698; LIB045499_CHS00173890_S2_L003, 2136062; LIB045499_CHS00173890_S2_L004, 2067419; LIB045499_CHS00173891_S3_L001, 1492088; LIB045499_CHS00173891_S3_L002, 1456747; LIB045499_CHS00173891_S3_L003, 1532881; LIB045499_CHS00173891_S3_L004, 1461992; LIB045499_CHS00173892_S4_L001, 2522360; LIB045499_CHS00173892_S4_L002, 2480676; LIB045499_CHS00173892_S4_L003, 2588428; LIB045499_CHS00173892_S4_L004, 2491652; LIB045499_CHS00173893_S5_L001, 1988063; LIB045499_CHS00173893_S5_L002, 1943597; LIB045499_CHS00173893_S5_L003, 2034599; LIB045499_CHS00173893_S5_L004, 1950723; LIB045499_CHS00173894_S6_L001, 2170419; LIB045499_CHS00173894_S6_L002, 2109665; LIB045499_CHS00173894_S6_L003, 2223329; LIB045499_CHS00173894_S6_L004, 2119886; LIB045499_CHS00173895_S7_L001, 3105918; LIB045499_CHS00173895_S7_L002, 3047315; LIB045499_CHS00173895_S7_L003, 3181092; LIB045499_CHS00173895_S7_L004, 3058192; LIB045499_CHS00173896_S8_L001, 3799882; LIB045499_CHS00173896_S8_L002, 3746522; LIB045499_CHS00173896_S8_L003, 3891180; LIB045499_CHS00173896_S8_L004, 3765473; Length of reads: 150 bp; paired-end

Antibodies

IgG antibody as included in SimpleChIP Plus Enzymatic Chromatin IP Kit (Cell Signaling Technology; #9005), anti-Flag (Sigma, F3165)

Peak calling parameters

```
# Specify the command line program and parameters used for read mapping and peak calling,
# including the ChIP, control, and index files used

# the command line program and parameters are the same for each experiment

# read mapping
bowtie2 -p 16 -q --local \
	-x /n/groups/flyrnai/yifang/ref/Drosophila_melanogaster/UCSC/dm6/Sequence/Bowtie2Index/genome \
	-1 /n/groups/flyrnai/yifang/Projects/With/Hongwen/ChIP-seq/2020-03-16_ChIP-seq/trim_output/
LIB045499_CHS00173889_S1_L001_R1_val_1.fq \
	-2 /n/groups/flyrnai/yifang/Projects/With/Hongwen/ChIP-seq/2020-03-16_ChIP-seq/trim_output/
LIB045499_CHS00173889_S1_L001_R2_val_2.fq \
	-S /n/groups/flyrnai/yifang/Projects/With/Hongwen/ChIP-seq/2020-03-16_ChIP-seq/bowtie2_output/
LIB045499_CHS00173889_S1_L001.sam

# peak calling
macs2 callpeak \
-t bowtie2_output/LIB045499_CHS00173894_S6_unique.bam \
-c bowtie2_output/LIB045499_CHS00173891_S3_unique.bam \
-B --SPMR \
-f BAMPE \
-g dm \
-n odj_vs_control \
--outdir macs2_output 2> macs2_output/odj_vs_control.log &
```

Data quality

Peaks at 5% FDR and above 5-fold enrichment: dwg_vs_control, 1943; dwg_vs_input, 1831; odj_vs_control, 1202

Software

TrimGalore 0.6.4, bowtie2 2.3.5.1, SamTools 1.6, MACS2 2.2.6, Homer 4.11, DeepTools 3.4.0
